# Alcohol-dependent downregulation of apolipoprotein H exacerbates fatty liver and gut microbiota dysbiosis in mice

**DOI:** 10.1186/s12944-022-01699-7

**Published:** 2022-09-19

**Authors:** Yaming Liu, Zhe Wu, Yong Zhang, Binbin Chen, Shuqi Yu, Wanyun Li, Jianlin Ren

**Affiliations:** 1 Department of Gastroenterology and Hepatology, Xiamen University Zhongshan Hospital, Xiamen, 361001 Fujian Province China; 2 Department of Digestive Diseases, School of Medicine, Xiamen University, Xiamen, 361001 Fujian Province China; 3 Digestive Department, Peking University People’s Hospital, Beijing, 100001 China; 4 School of Life Sciences, Xiamen University, Xiamen, 361001 Fujian Province China; 5 School of Medicine, Xiamen University, Xiamen, 361001 Fujian Province China; 6 Department of Pathology, Xiamen University Zhongshan Hospital, Xiamen, 361001 Fujian Province China

**Keywords:** Apolipoprotein H, Alcohol-related liver disease, Fatty liver, Lipid metabolism, Gut microbiota

## Abstract

**Background:**

Alcohol-related liver disease (ALD) is a major chronic liver ailment caused by alcohol overconsumption and abuse. Apolipoprotein H (APOH) participates in lipid metabolism and might have a potential regulatory role in ALD. Therefore, this study aimed to explore the effects of ApoH on alcohol-induced liver injury and gut microbiota dysbiosis.

**Methods:**

*ApoH*^*−/−*^ mice were generated and the synergic alcoholic steatohepatitis mouse model was constructed, which were used to assess liver function and pathological changes.

**Results:**

*ApoH*^*−/−*^ mice clearly exhibited spontaneous steatohepatitis. Severe hepatic steatosis was observed in alcohol-fed WT and *ApoH*^*−/−*^ mice, in which ApoH expression was reduced post alcohol consumption. Moreover, RNA-seq and KEGG pathway analyses indicated that differential expression genes enriched in lipid metabolism and oxidation–reduction process between in alcohol-fed *ApoH*^*−/−*^ mice and pair-fed control mice. Finally, gut microbiota diversity and composition were assessed by 16S rRNA Illumina next-generation sequencing. Alpha diversity of enterobacteria was lower in *ApoH*^*−/−*^ mice with ethanol feeding than in ethanol-fed WT mice and all control-fed mice (*P* < 0.05). Moreover, KEGG enrichment analysis, using PICRUSt software, revealed that metabolic functions were activated in the gut microorganisms of *ApoH*^*−/−*^ mice with ethanol feeding (*P* < 0.05).

**Conclusions:**

Alcohol-downregulated *ApoH* expression, leading to the progress of fatty liver disease and gut microbiota dysbiosis.

## Introduction

Alcohol abuse and overconsumption are increasing worldwide, which contributes to alcohol-related liver disease (ALD) [1, 2]. The pathogenic factors of ALD are associated with alcohol metabolism, gender, genetic and environmental factors, diet, and the microbiome. Metabolic disruption and gut dysbiosis are the major pathophysiologic mechanisms of ALD and play a prominent function in the progression of alcohol associated liver injury [3–6]. Therefore, there is an urgent need to identify clinical unmet needs in ALD and clarify the precise mechanism underlying ALD pathogenesis.

Apolipoprotein H (APOH) is an abundant plasma apolipoprotein primarily produced in the liver [7, 8]. The previous study was focused on the function of apolipoprotein H (APOH) during hepatitis B infection [9, 10]. However, APOH has been known to bind with specific to lipoproteins and activate lipoprotein lipase during triglyceride (TG) metabolism, and is closely associated with lipid metabolism [11–17]. APOH isoforms are associated with the expression levels of TG, total cholesterol (TC), high-density lipoprotein cholesterol (HDL-C), apolipoproteins B (APOB) and apolipoproteins A (APOA) [18–20]. Together, it is interesting to explore the potential regulatory mechanism of APOH in alcohol-associated fatty liver disease in this study.

In the current study, the interactions across ApoH-associated alcohol metabolism, fatty liver, and gut microbiota are focused to define potential pathogenesis of alcohol-related liver injury. The specific experiments targeting on gut microbiome, metabolic dysbiosis and binge drinking-induced liver injury, are performed using a murine model.

## Methods

### Construction of *ApoH* gene knockout mouse model by clustered regularly interspaced short palindromic repeats (CRISPR)

The 8–10-week-old wild type (WT) C57BL/6 J mice were purchased from Xiamen University Laboratory Animal Center (Xiamen, China). Five mice were placed in a single cage and raised in a proper sterile environment (temperature: 20–26 °C, humidity: 40%–70%).

C57BL/6 J females were superovulated by injecting 6.5 U of pregnant mare serum gonadotropin (Sigma, Burlington, MA, USA), subsequently by 6.5 U of human chorionic gonadotropin (Sigma). Post-injection 48 h, the above mice were then mated with C57BL/6 J males overnight. The APOH small base-pairing RNA (50 ng/μL; sequences being 5′-TCCAAAGTTTGCACTCCTTA-3′ and 5′-GATTGCCAGAATGCCTGGGT-3′) were injected into the embryos and cultured in M16 medium (Sigma) at 37 °C in 95% air and 5% CO_2_ for producing mutant mice. The mutant mice mated with wild type C57BL/6 J mice, and their offspring were then crossed to produce *ApoH*^−/−^ mice. The *ApoH*^−/−^ mice had a 140 bp net deletion in transcriptional X1 exon 3. The knockout gene was identified by polymerase chain reaction (PCR).

### Construction of Chronic-binge ethanol feeding mouse model

8–10-week-old WT and *ApoH*^*−/−*^ C57BL/6 female mice were used to construct chronic-binge ethanol feeding model (the NIAAA model). The process of the model construction includes chronic ethanol feeding (oral feeding of the Lieber-DeCarli ethanol liquid diet for 10 d *ad libitum*) plus a single binge ethanol feeding [21–23]. To purchase the Lieber-DeCarli alcoholic liquid feed (no. TP4030D) and control liquid feed (no. TP4030C) from Tropical Animal Feed High-Tech Co. Ltd., Nantong City, Jiangsu Province, China. The *ApoH*^*−/−*^ and WT mice were fed with control or ethanol diets daily (each group: *n* = 10).

### Assay of serum alanine aminotransferase (ALT) and aspartate aminotransferase (AST) levels in mice

Mouse peripheral blood was collected from the eyeball and centrifuged at 3,000 rpm for 15 min at room temperature (approximately 23–25 °C). The serum samples were then stored at − 80 °C until required. Serum ALT and AST levels were quantified by commercial kits (Cat# 105–000442-00 and Cat# 105–000443-00, Mindray, Shenzhen, China) using a Mindray BS-240vet fully automatic biochemical analyzer.

### Histopathological analysis

To determine macroscopic changes in tissue structure, 4% paraformaldehyde-fixed, paraffin-embedded mouse liver tissues were stained with hematoxylin and eosin (H&E). The method of Oil Red O staining was used to detect neutral triglycerides and lipids in liver sections using a commercial staining kit (ab150678; Abcam, Cambridge, UK). The slides were then observed under a fully automatic biological microscope (BA600-4, Motic, Xiamen, China).

### Liver triglyceride (TG) assay

Mouse liver sections were collected and flash-frozen in liquid nitrogen. Thereafter, the frozen liver tissues were weighed and homogenized in absolute ethanol. Triglyceride content was quantified using a commercial testing kit (Cat# A110-1–1, Nanjing Jiancheng Bio-engineering Institute, Nanjing, China) according to the manufacturer’s instructions.

### RNA sequencing and analysis

RNA was isolated from the livers of respective treated groups using the detailed real-time quantitative PCR (RT-qPCR) procedures reported previously [9]. The used primers were listed in Table 1. Frozen live tissues were randomly selected (each group; *n* = 3) and sent to the Beijing Genomics Institute (BGI) for transcriptome sequencing, and BGI automatic analysis software was performed to analyze the RNA sequencing data. In KEGG pathway analysis, the enrichment ratio calculated using the Term Candidate Gene Num / Term Gene Num, was represented in the *x*-axis, whereas the KEGG pathway was represented in the *y*-axis. The Q-value was obtained from FDR correction of the *P-*value. A Q-value ≤ 0.05 represented significant enrichment.

**Table 1:** Mouse primers in RT-qPCR analysis

Gene	Primers (5ʹ-3ʹ)	
GAPDH	Forward	AACGACCCCTTCATTGAC
	Reverse	TCCACGACATACTCAGCAC
APOH	Forward	TGGCATTGAACTCACACT
	Reverse	GAATGTTCCTGGCAGTTG
CYP1A1	Forward	CAGGCTGACTCTGAACTTGC
	Reverse	AGACCAAGAGCTGATGCAGT
CYP1B1	Forward	CACTATTACGGACATCTTCGG
	Reverse	AGGTTGGGCTGGTCACTC
CYP2A5	Forward	CCAAGAAAGTGGAACACAATCA
	Reverse	GGGGTTCTTCTTCTCCTCCA
CYP2B10	Forward	TTTCTGCCCTTCTCAACAGGAA
	Reverse	ATGGACGTGAAGAAAAGGAACAAC
CYP2E1	Forward	CGTTGCCTTGCTTGTCTGGA
	Reverse	AAGAAAGGAATTGGGAAAGGTCC
CYP11A1	Forward	AAGTATGGCCCCATTTACAGG
	Reverse	TGGGGTCCACGATGTAAACT
CYP26A1	Forward	ACTTACCTAGGACTCTACCCAC
	Reverse	GCTGTTCCAAAGTTTCCATGTC

### 16S rRNA gene quantitative analysis

Stool samples were collected from mice and stored at − 80 °C. The samples were subsequently sent to Majorbio Corporation (Shanghai, China) for microbial diversity determination. Samples from the same cages were pooled in equal concentrations at different time points, and amplicons were sequenced on an Illumina HiSeq platform (Illumina, San Diego, CA, USA). The V3–V4 region of 16S rRNA gene was used as the bacteria-specific fragment with primers 338F (5ʹ-ACTCCTACGGGAGGCAGCAG-3ʹ) and 806R (5ʹ-GGACTACHVGGGTWTCTAAT-3ʹ). Majorbio automatic analysis software was used for sequencing data analysis.

### Function prediction using PICRUSt

Using the above 16S sequencing data, to explore the microbial community composition to predict their function using KEGG enrichment analysis in PICRUSt software (version 1.1.0).

### Statistical analysis

Experimental results are expressed as mean ± standard error of the mean (SEM) from three or more independent experiments. Statistical significance was assessed using Student’s *t*-test for comparisons between two groups or using ANOVA for comparisons across more than two groups. Differences were considered statistically significant at *P* < 0.05.

When performing principal component analysis (PCA), the plot of primary features was prepared using STAMP software based on multiple group analysis, and ANOVA was conducted to determine the differences between groups. A Games–Howell post-hoc test was performed to determine confidence intervals, and Benjamin–Hochberg FDR was used to correct for multiple comparisons.

## Results

### Construction of chronic-binge ethanol feeding mouse model using ***ApoH***^***−/−***^ C57BL/6 and WT mice

**Fig. 1:**
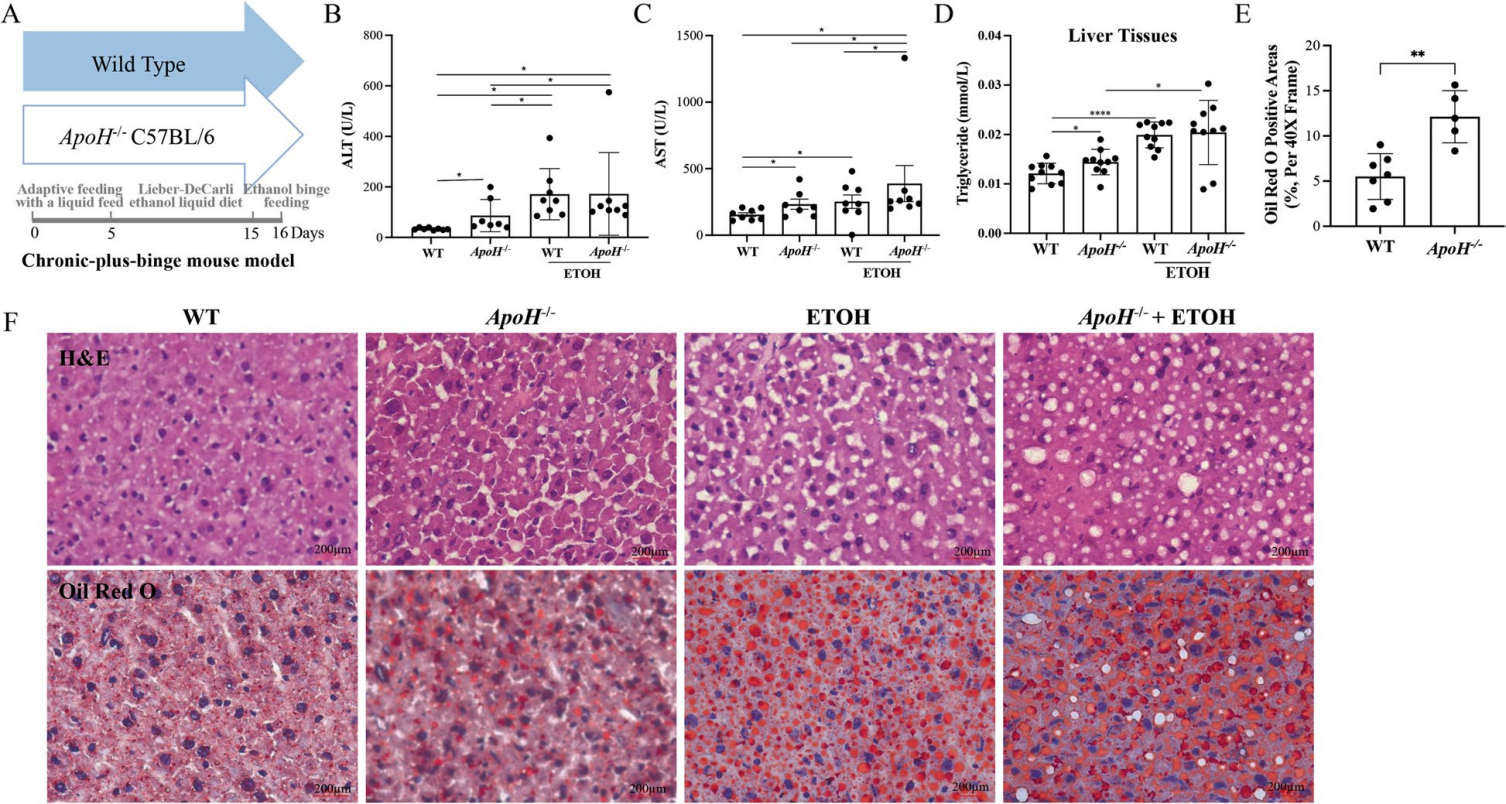
Construction of chronic-binge ethanol feeding mouse model using C57BL/6 *ApoH*^*−/−*^and WT mice. **A** Image of the mouse model of chronic-plus-binge ethanol feeding. **B**, **C** Estimation of serum ALT and AST levels in mice. **D** Liver triglyceride content in wild type (WT), *ApoH* knockout (KO), alcohol-fed WT, and alcohol-fed KO mice, normalized to liver protein content. **E** Quantitative analysis of the stained liver tissues. The percentage of steatosis (%) calculated as the ratio of steatosis area to liver parenchyma area. **F** Hematoxylin and eosin (H&E) staining and Oil Red O staining of liver tissues (40x). Results are presented as mean ± SEM. *P < 0.05

The *ApoH*^*−/−*^ mouse model was used to explore the potential function of *ApoH* in the liver. In this study, a mouse model of chronic-plus-single binge ethanol feeding was constructed using C57BL/6 *ApoH*^*−/−*^ and WT mice (Fig. 1A). Liver function parameters were analyzed based on ALT and AST levels, which were significantly increased in *ApoH*^*−/−*^ mice than in WT mice fed the control diet (*P* < 0.05; Fig. 1B and C). AST levels were remarkably elevated in *ApoH*^*−/−*^ mice fed the ethanol diet than in knockout mice pair-fed the control diet (*P* < 0.05; Fig. 1B and C). Liver triglyceride content also increased significantly in *ApoH*^*−/−*^ mice than in WT mice under control diet (*P* < 0.05), whereas it was significantly increased in WT and *ApoH*^*−/−*^ mice when both were fed ethanol (*P* < 0.05; Fig. 1D). Further, the degree of steatosis was evaluated in the stained liver tissue (Fig. 1E). Hepatic steatosis was observed in normal diet-fed *ApoH*^*−/−*^ mice, whereas severe steatosis was observed in alcohol-fed WT and *ApoH*^*−/−*^ mice (Fig. 1F). These findings suggested that absence of the *ApoH* gene induces hepatic steatosis, which is exacerbated by ethanol consumption.

### RNA-seq analysis of liver sections in the chronic-binge ***ApoH***^−/−^ mouse model highlighted the role of metabolic pathways

**Fig. 2:**
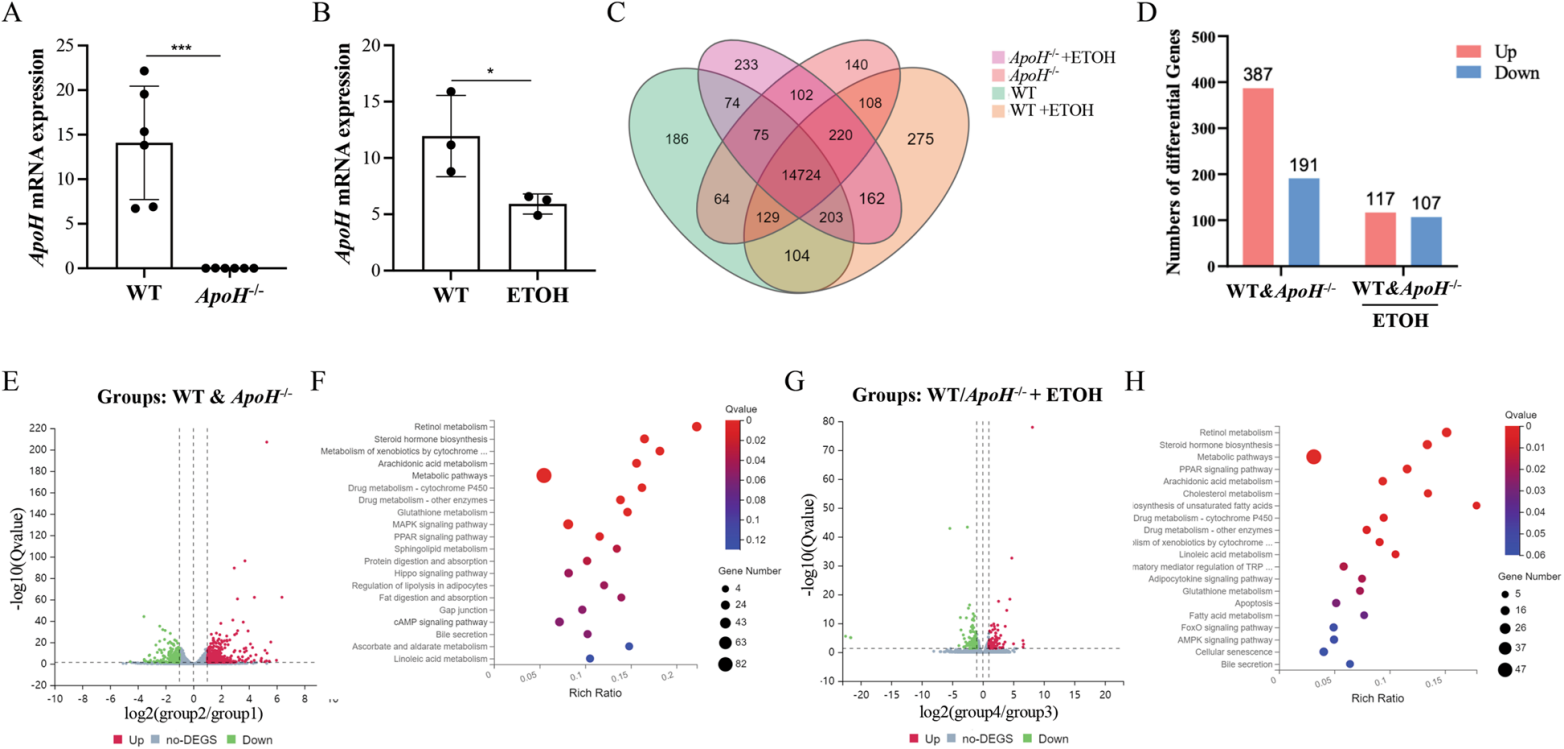
RNA-seq analysis of liver sections of the chronic-binge *ApoH*^−/−^ mouse model. **A**, **B** *ApoH* mRNA levels assayed using qPCR; *P < 0.05, ***P < 0.001. **C** Venn map showing the extent of gene expression in the four different groups. **D** The number of differentially expressed genes in WT and *ApoH*^*−/−*^ mice and their alcohol-fed counterparts. **E** Volcano plot showing the DEGs in WT and *ApoH*^*−/−*^ mice. **F** KEGG pathways analysis depicting gene richness in *ApoH*^*−/−*^ mice compared with that in the WT control group. **G** Volcano plots of the DEGs in WT and *ApoH*^*−/−*^ mice fed alcohol. **H** Bubble diagram representing gene abundance in alcohol-fed mice, including *ApoH*^*−/−*^ mice, versus the WT control group, using KEGG pathways analysis

To explore the underlying mechanism of alcohol induced liver injury using the alcoholic fatty liver mouse model (the NIAAA model), RNA-seq was performed on liver sections from Lieber-DeCarli alcohol diet-fed C57BL/6 WT and *ApoH*^*−/−*^ mice and their respective controls. The *ApoH* mRNA expression in *ApoH*^*−/−*^ mice and alcohol-fed WT mice are illustrated in Fig. 2A and B, respectively. The Venn diagram and UpSet plots illustrate the interactions between combinations of the four different groups while hiding those without interactions (Fig. 2C). Figure 2D shows the number of differential expression genes (DEGs) between the WT and *ApoH*^*−/−*^ mice and their alcohol-fed groups. Volcano plots further indicated the expression of differential genes in the various groups (Fig. 2E and G). Metabolic pathways were indicated to be predominant in KEGG signaling pathways (Fig. 2F and H). Among them, the DEGs between WT mice fed an alcohol diet and those fed paired control diet were predominantly associated with MAPK signaling pathway, retinol metabolism, and steroid hormone biosynthesis (Top 3). In contrast, the DEGs between *ApoH*^−/−^ mice fed an alcohol diet and the ones fed paired control diet were predominantly enriched in retinol metabolism, steroid hormone biosynthesis, and PPAR signaling pathway (Top 3).

**Fig. 3:**
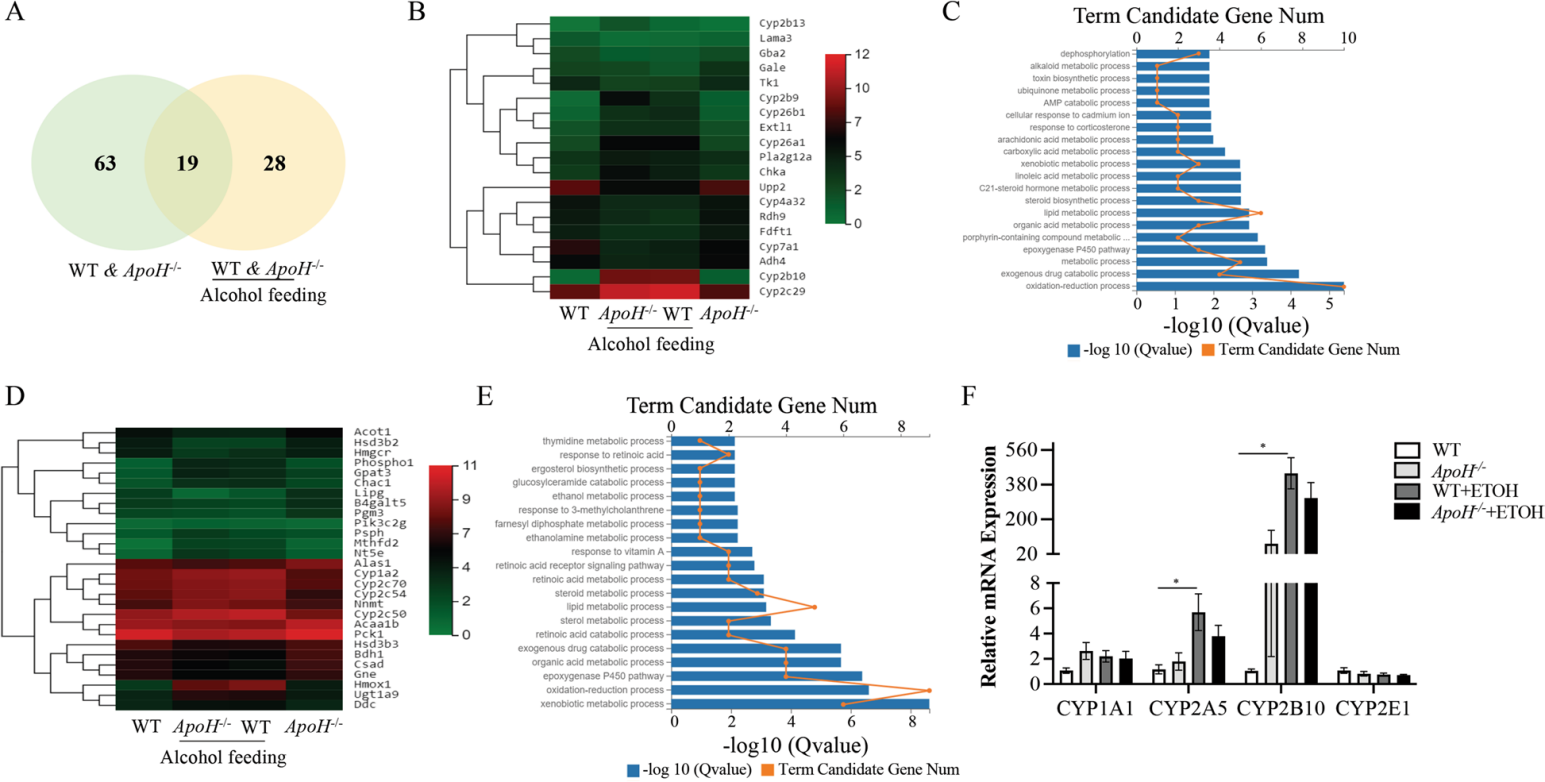
Differential gene expression obtained via RNA-seq data. **A** Number of DEGs between control-fed WT and *ApoH*^*−/−*^ mice and their alcohol-fed counterparts, and the relationship between them. **B** Heatmap representing the 19 common DEGs between the compared groups. **C** DEGs abundance in *ApoH*^*−/−*^ mice versus the WT control group analyzed using KEGG pathways. **D** Heatmap representing the 28 DEGs between WT and *ApoH*^*−/−*^ mice post alcohol feeding. **E** Column diagram representing the abundance of DEGs in alcohol-fed and control *ApoH*^*−/−*^ as well as WT groups analyzed using KEGG pathways. **F** Relative expression of genes involved in regulating CYP450 enzymes in WT and *ApoH*^*−/−*^ mice given alcohol and control diets. Values are represented as the mean ± SEM (*n* = 3). *P < 0.05

Subsequently, the number of DEGs was summarized in the different groups (Fig. 3A). A total of 82 DEGs were identified between the control-fed WT and *ApoH*^*−/−*^ mice and 47 DEGs were identified between alcohol-fed WT and *ApoH*^*−/−*^ mice. A comparison of gene expression profiles across the alcohol-fed groups led to the identification of 19 DEGs (Fig. 3B). Further, KEGG pathway analysis indicated that oxidation–reduction and lipid metabolic processes are most enriched in the signaling pathways (Fig. 3C). The remaining 28 DEGs between alcohol-fed WT and *ApoH*^*−/−*^ mice were analyzed, and Fig. 3D illustrates the detailed information using a heatmap. The functional genes were enriched in oxidation–reduction and lipid metabolic processes, based on KEGG pathway analysis (Fig. 3E). Some cytochrome P450 (CYP) enzyme-related genes were identified among the 47 DEGs.

CYP450 enzymes act as a terminal oxidase in the multi-function oxidase system to metabolize different endogenous substrates and xenobiotics. The isoenzymes CYP2E1 and CYP2A5 primarily participate in alcohol metabolism and alcohol-induced liver injury [24–26]. The changes in expression of certain CYP450 genes were examined, namely, *Cyp1a1, Cyp1b1, Cyp2a5, Cyp2b10, Cyp2e1, Cyp11a1*, and *Cyp26a1* (Fig. 3F). The mRNA levels of *Cyp2a5* and *Cyp2b10* were significantly increased in alcohol-fed WT mice than in normal diet-fed WT controls (*P* < 0.05). However, no significant difference was observed between the two *ApoH*^*−/−*^ groups. The expression of *Cyp1b1, Cyp11a1*, and *Cyp26a1* was undetectable in these samples. Taken together, the results suggested that ApoH downregulation primarily impacts liver lipid metabolism, which is mainly associated with CYP450 enzyme-activated pathways.

### Changes in community diversity and bacterial species abundance in the chronic-binge *ApoH*^−/−^ mouse model

**Fig. 4:**
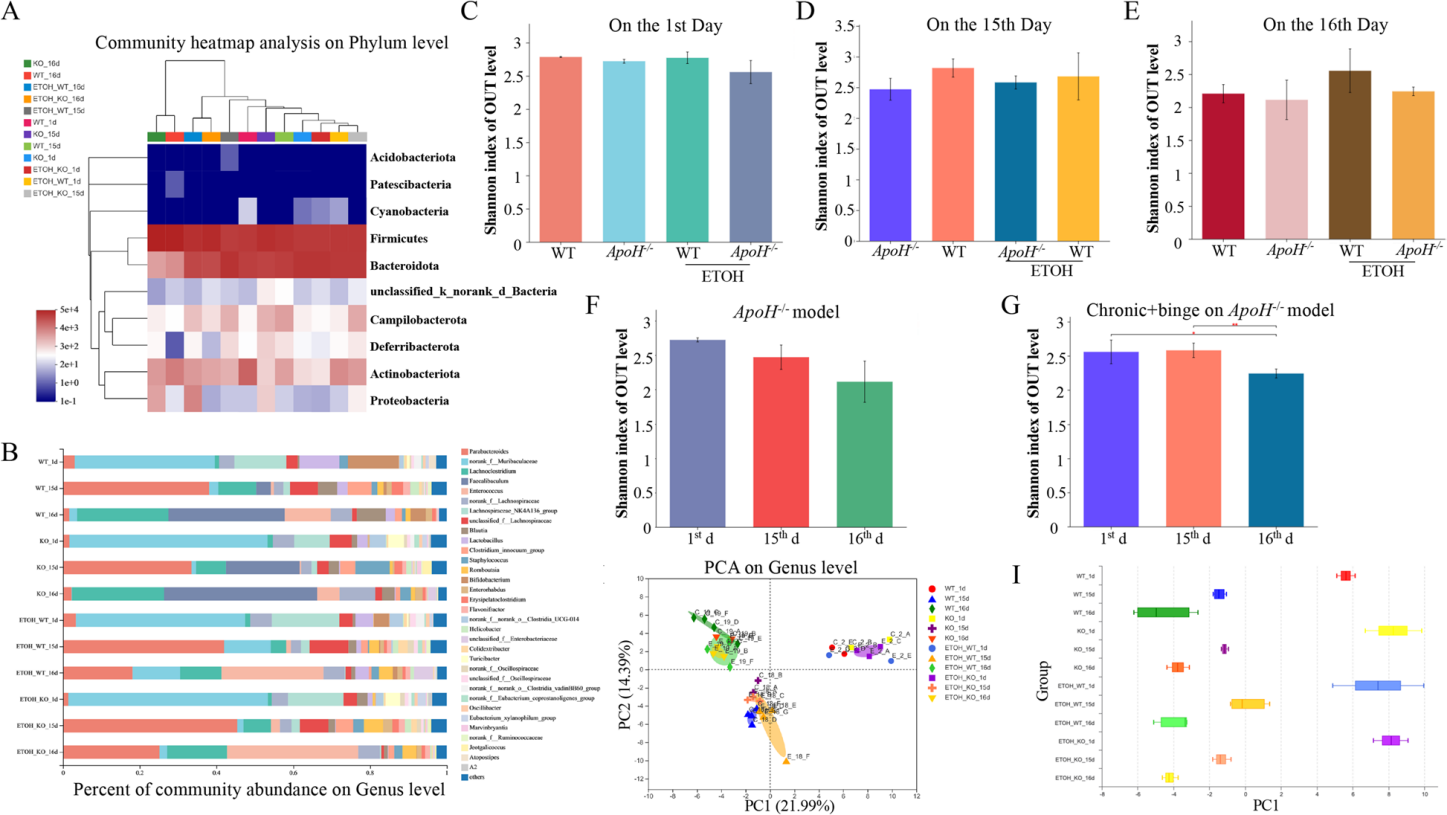
Changes in community diversity and bacterial species abundance in the chronic-binge *ApoH*^−/−^ mouse model. **A**, **B** Bacterial composition at phylum (A) and genus (B) levels in WT and *ApoH*^*−/−*^ mice fed alcohol and their pair-fed controls. **C-E** Alpha diversities of WT and *ApoH*^*−/−*^ mice and their alcohol-fed counterparts were analyzed and displayed using the Shannon index of OTU level temporally (*P* > 0.05). **F**, **G** Alpha diversity of enterobacteria from *ApoH*^*−/−*^ mice after 15-day chronic alcohol feeding (F) and alcohol binging (G). **H**, **I** Beta diversity of WT and *ApoH*^*−/−*^ mice fed an alcohol diet and their pair-fed controls, where (H) shows the PCA plot, and (I) shows the boxplot representing the distribution of samples in different groups along the PC1 axis

Recently, the gut-liver axis has been deemed one of the main regulatory factors in ALD [3, 4, 27]. Therefore, to explore the gut community diversity and differences in bacterial species abundance between alcohol- or control diet-fed WT and *ApoH*^*−/−*^ mice. The total bacterial composition and abundance were remarkably reduced in WT and *ApoH*^*−/−*^ mice fed an alcohol diet. However, at the phylum level, the abundance of Bacteroidetes increased in WT and *ApoH*^*−/−*^ mice after 10-day chronic ethanol feeding, whereas that of Firmicutes increased sharply after the whole chronic-binge ethanol feeding. Abundance of Actinobacteria was lower in *ApoH*^*−/−*^ mice than in WT mice fed either alcohol or control diet (Fig. 4A). In the beginning of adaptive liquid diet feeding in *ApoH*^*−/−*^ mice, the abundance of *norank_f_Muribaculaceae*, *Lachnospiraceae*, *Clostridia*, and *Turicibacter* increased, whereas that of *Lactobacillus* and *Bifidobacterium* decreased. However, at the end of chronic-binge control diet feeding, the abundance of *Lachnoclostridium* and *Parabacteroides* increased, whereas that of *Romboutsia*, *Lactobacillus*, and *Bifidobacterium* decreased in *ApoH*^*−/−*^ mice than in WT mice (Fig. 4B).

Thereafter, alpha diversity was analyzed, and Shannon index for the OTU level was used as a metric/index (Fig. 4C–E). No significant difference was observed in alpha diversity between WT and *ApoH*^*−/−*^ mice administered the alcohol and pair-fed control diets at three different time points, namely the first day, chronic 10-day feeding, and final binge intake (Shannon index: *P* > 0.05). No significant difference was observed in *ApoH*^*−/−*^ mice at the three different time points (Fig. 4F). However, at the end of the construction of chronic-binge model, alpha diversity of enterobacteria from *ApoH*^*−/−*^ mice was lower than that corresponding to mice on the first day and to mice after chronic 10-day ethanol feeding (*P* < 0.05; Fig. 4G). Additionally, beta diversity and PCA indicated the bacterial community structure to be segregated differently between the four groups at the genus level (Fig. 4H and I).

### Alcohol feeding-dependent ApoH downregulation impacted metabolic functions of gut microbiota

**Fig. 5:**
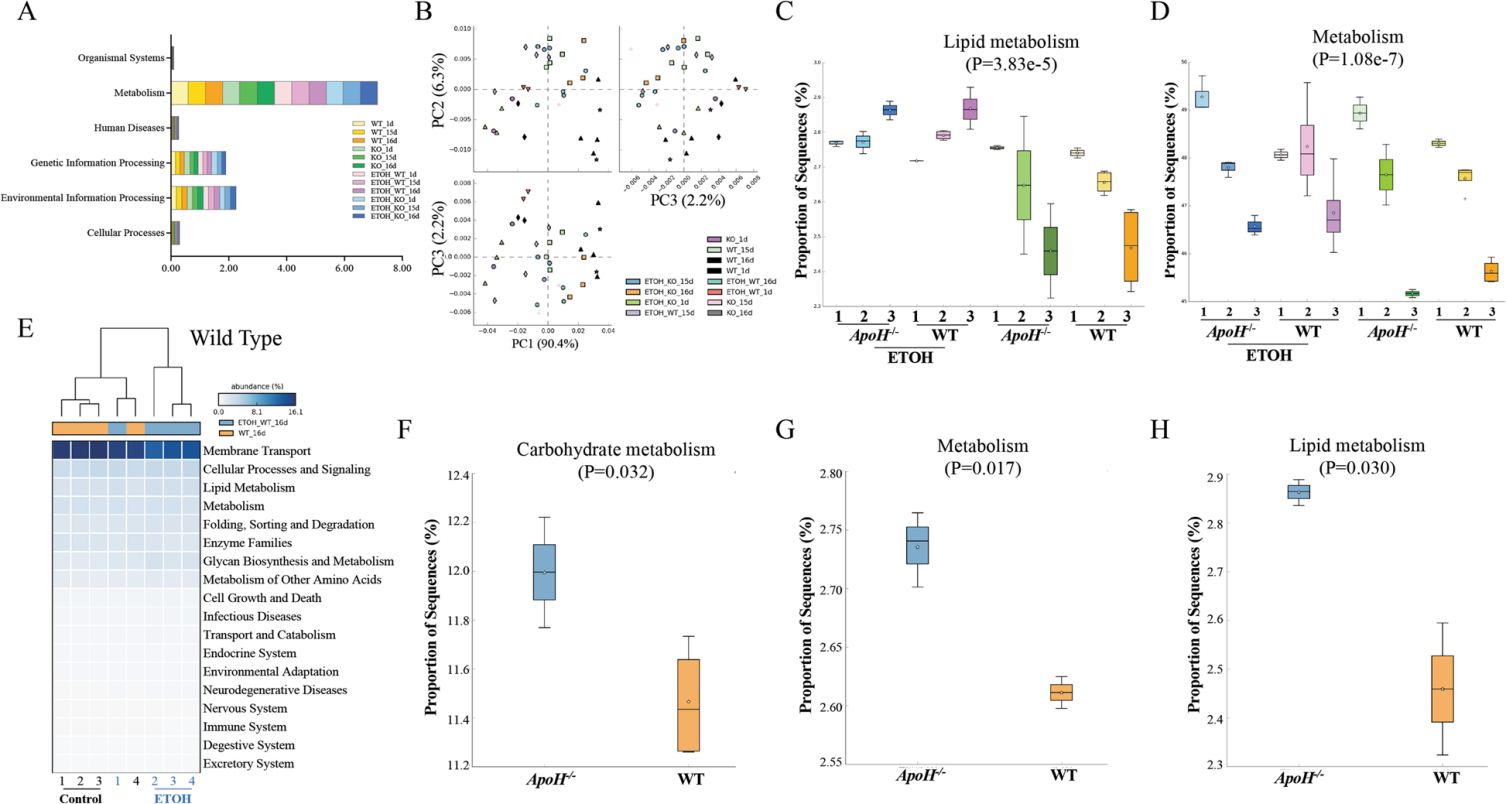
PICRUSt recapitulates biological findings from the chronic-binge *ApoH*^−/−^ mouse model. **A** The functional profiling of microbial communities was predicted using the PICRUSt KEGG pathway. **B**-**H** Analysis of the sequencing data using STAMP software to predict potential metabolic differences. **B** Principal component analysis (PCA) plot using STAMP software. The *x* and *y* axes indicate principal components 1, 2, and 3 accounting for 85.2%, 9.0%, and 2.8% of the total variation in predicted functions, respectively. **C**, **D** Distribution of relative abundance of “metabolism” and “lipid metabolism” between different groups (*P* < 0.05). **E** Heatmap illustrating the abundance of selected active KEGG pathways in WT mice given alcohol and the pair-fed control diet. **F** Carbohydrate metabolism shown as the active KEGG pathways in WT and *ApoH*^*−/−*^ mice with alcohol feeding (*P* = 0.032). **G**, **H** Metabolism and lipid metabolism pathways were activated and differed significantly between *ApoH*.^*−/−*^ mice fed alcohol and control diets (*P* = 0.017 and *P* = 0.030, respectively)

The different microbial community profiles was explored to predict their function using KEGG enrichment analysis in PICRUSt software. The results indicated that the gut microbiota primarily performed metabolic functions in the different groups; however, no significant difference was observed between them (Fig. 5A). The sequencing data was further analyzed using STAMP software to predict potential metabolic differences. The principal components 1, 2, and 3 accounted for 90.4%, 6.3%, and 2.2% of the total variation in the predicted functions, respectively (Fig. 5B). It was proceeded to analyze the relative abundance and distribution of metabolism and lipid metabolism in the different groups. Significant differences were observed across the different groups, as illustrated in Fig. 5C and D (*P* < 0.05). The heatmap indicated abundance of the selected active KEGG pathways in WT mice fed either an alcohol or control diet (Fig. 5E). From the selected KEGG pathway, numerous active signaling pathways were identified, including metabolism, lipid metabolism, glycan biosynthesis and metabolism, and cell growth and death, in WT mice fed either alcohol or control diet. The carbohydrate metabolism pathway was more active in alcohol-fed *ApoH*^*−/−*^ mice than in alcohol-fed WT mice (*P* = 0.032) (Fig. 5F). Moreover, “metabolism” and “lipid metabolism” signaling pathways were more highly activated in alcohol*-*fed *ApoH*^*−/−*^ mice than in control diet-fed *ApoH*^*−/−*^ mice (*P* = 0.017 and *P* = 0.030; Fig. 5G and H, respectively). In conclusion, ApoH downregulation could affect the metabolic regulation of gut microbiota in mice, leading to changes in the metabolic outcome/pathways.

## Discussion

**Fig. 6:**
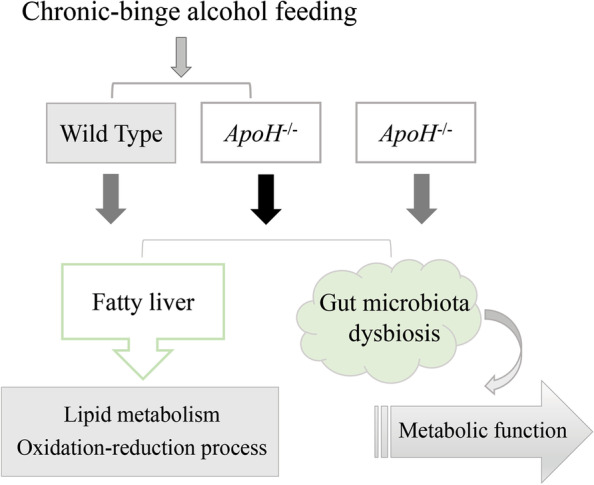
Model representing the function of ApoH in alcohol-induced liver injury. In the chronic and binge ethanol mouse models, alcohol downregulated ApoH expression to aggravate fatty liver and gut microbiota dysbiosis. Specifically, alcohol downregulated ApoH expression to activate the oxidation–reduction process, particularly the CYP450 enzymes system, and induced dysbiosis of lipid metabolism in ALD. The downregulation of ApoH caused an imbalance in the intestinal micro-ecological environment and resulted in functional metabolic disorders of the gut microbiota in mice

Alcohol-related liver disease is widely believed to be related to human alcohol metabolism, gender, genetic and environmental factors, diet, and microbiome. However, the metabolic disruption and gut dysbiosis, especially affecting bile acid signaling and metabolism, play a prominent role in disease progression in ALD [5]. The present study was investigated the effects of ApoH on chronic and binge ethanol-induced liver injury and gut microbiota dysbiosis. These results were found that (1) *ApoH*^*−/−*^ mice developed spontaneous steatohepatitis, and those fed an alcohol diet had severe hepatic steatosis, and (2) alcohol downregulated ApoH expression in the liver, activating metabolic pathways, promoting hepatocyte steatosis, and inducing gut microbiota dysbiosis in mice (Fig. 6).

Our mouse model of alcoholic hepatitis in an *ApoH*^−/−^ background highlighted the impact of alcohol on liver dysfunction. However, *ApoH*^−/−^ mice exhibited spontaneous steatohepatitis, whereas those fed alcohol had more severe fatty liver hepatitis than WT mice fed alcohol. In addition, we found alcohol-induced downregulation of *ApoH* expression to similarly result in compromised liver function and steatohepatitis development. Therefore, it was speculated the decreased APOH-mediated metabolic dysbiosis to possibly be an independent or alcohol metabolism-associated pathogenic factor in the process of fatty liver formation. The future prospect would be to clarify the potential regulatory mechanism of APOH expression in ALDs and NAFLDs.

Functional analysis of sequencing data from liver tissues indicated that the predominantly enriched DEGs are strongly associated with metabolic signaling pathways. The DEGs were categorized in different groups and identified various CYP450 enzyme-related genes. CYP enzymes serve as membrane-bound proteins to modulate cellular metabolism, homeostasis, and intoxication and detoxification of xenobiotics [28]. The substrates of various xenobiotics and endogenous can activate CYP enzymes at transcriptive level through receptor-dependent signaling pathways [28]. Moreover, CYP constitutes a major component of the microsomal ethanol-oxidizing system (MEOS), which in turn contributes to ALD [24, 26]. In the present study, KEGG pathway analysis indicated that the functional genes are enriched in processes such as oxidation–reduction and lipid metabolism. It was found that *Cyp2a5* and *Cyp2b10* mRNA expression significantly increased in WT mice fed an alcohol diet than in WT mice fed a normal diet. However, *Cyp1b1*, *Cyp11a1*, and *Cyp26a1* were undetectable in the samples. CYP2E1 is a pathogenetic factor in alcohol-induced oxidative liver injury; however, CYP2A5 protects against ALD, which might be associated with nuclear factor erythroid 2-related factor 2 (Nrf2)-mediated antioxidant effects. Moreover, CYP2B10 expression has been shown to be increased by activators of Nrf2, such as phorone (an Nrf2 activator) and phenobarbital (a classical CYP2B inducer), in wild type but not in *Nrf2*^−/−^ mice [24, 25, 29]. Considering the above findings, another prospect for future studies would be to combine single-cell sequencing data with spatial information for accurate and directed insights into ApoH-mediated disruption of metabolic homeostasis in alcohol- and nonalcohol-related fatty liver.

Recently, researchers have paid much attention to the role of gut-liver axis in the pathophysiological processes of chronic liver diseases. The gut and the liver interact via multiple pathways. Bile acids (BAs) are known to interact closely with the host and microbiome, and modulate microbiota, microbial metabolites, and shifts in primary and secondary BA profiles [4, 30, 31]. Moreover, recent understanding of nuclear farnesoid X (FXR) and membrane Takeda G protein-coupled receptor 5 (TGR5) signaling pathways had enhanced focus on the impact of crosstalk of gut, bile acid and liver on liver pathology. Bile acid-based molecular mechanism had shown exciting results in addressing liver inflammation and fibrosis [32–34]. Additionally, activation of some CYP450 enzymes has been shown to be closely associated with bile acid metabolism [34].

In the current study, the data indicated the total bacterial composition and abundance to be significantly reduced in alcohol-fed WT and *ApoH*^*−/−*^ mice. The findings on the alpha diversity of enterobacteria corroborated those of previous studies [3, 35, 36]. The gut microbiota was observed to perform metabolic functions in all four different groups. Several active signaling pathways were observed in alcohol diet-fed WT mice, confirming the role of metabolism in maintaining hepatic homeostasis involving various pathways. Thus, downregulation of ApoH might aggravate alcohol-induced disorders of the intestinal micro-ecological environment and activate the metabolic functions of gut microbiota, especially with respect to carbohydrate and lipid metabolism. Future studies should focus on determining whether and how the dynamic gut microbiota modulate alcoholic fatty liver or ApoH-mediated fatty liver via the gut-liver axis.

### Comparisons with other studies and what the current work adds to the existing knowledge

The current study provided the following new information to the existing knowledge: a) *ApoH*^*−/−*^ mice clearly exhibited spontaneous steatohepatitis, b) by regulating the CYP450 enzyme signaling pathway, c) and via concomitant gut microbiota dysbiosis.

### Study strengths and limitations

The current study has several strengths. It is the first to characterize the alcohol-downregulated ApoH-induced lipid metabolic dysbiosis through CYP450 enzyme-activated pathways to attenuate steatohepatitis and cause disturbance of gut microbiota in mice. Several limitations of this study should also be acknowledged. First, effects of the downregulation of ApoH-mediated CYP450 signaling pathway were not experimentally clarified and only sequencing data were shown. Second, the interaction between downregulation of ApoH-induced metabolic activation and the dominant product of gut microbiota metabolism was not examined, and only 16S rRNA sequencing data were reported. Further studies should examine the accurate regulatory mechanism of ApoH-related lipid metabolism and concomitant gut microbiota metabolism in alcoholic and non-alcoholic fatty liver diseases.

## Conclusions

In summary, the above findings provided novel insights into the potential regulatory role of ApoH in fatty liver and gut microbiota dysbiosis, which led to a deeper understanding of the disease from gut-liver axis. Considering the downregulation of APOH might be an initial factor to break the balance of enterohepatic circulation, it is valuable to exploit a favorable treatment target for maintaining the metabolic balance of the body and bring a new therapeutic strategy for fatty liver disease.

## Data Availability

The liver-section RNA sequencing and stool 16S-rRNA sequencing were performed by Beijing Genomics Institute (BGI) and Shanghai Majorbio Corporation, respectively. The datasets generated and analyzed during the current study are available in the NCBI SRA repository (https://submit.ncbi.nlm.nih.gov/subs/sra/). All data related to this study are available upon request.

## Abbreviations

ALD: Alcohol-related liver disease
APOH: Apolipoprotein H
CRISPR: Clustered regularly interspaced short palindromic repeats
WT: Wild type
KO: Knockout
ALT: Alanine aminotransferase
AST: Aspartate aminotransferase
TG: Triglyceride
TC: Total cholesterol
HDL-C: High-density lipoprotein cholesterol
RT-qPCR: Real-time quantitative PCR
DEGs: Differentially expressed genes
PCA: Principal component analysis
CYP: Cytochrome P450
BA: Bile acid
FXR: Nuclear farnesoid X (FXR)
TGR5: Membrane Takeda G protein-coupled receptor 5
